# Linear IgA bullous dermatosis induced by nemvaleukin alfa, an engineered interleukin 2 molecule, in a patient with treatment-refractory metastatic melanoma

**DOI:** 10.1016/j.jdcr.2023.08.040

**Published:** 2023-09-14

**Authors:** Fatima Bawany, Vignesh Ramachandran, Eduardo Rodriguez, Randie H. Kim, Jeffrey S. Weber, Ian W. Tattersall

**Affiliations:** a Department of Dermatology, New York University, New York, New York; b Department of Medical Oncology, New York University, New York, New York

**Keywords:** interleukin-2, linear IgA bullous dermatosis, melanoma, nemvaleukin

## Introduction

Linear IgA bullous dermatosis (LABD) is characterized by linear deposition of IgA at the dermoepidermal junction. It is associated with medications, malignancies, and vaccinations.^1^ Here, we present a case of a patient with LABD treated with nemvaleukin alfa, a novel, engineered interleukin 2 (IL-2) receptor therapy currently in phase 2 investigation for treatment of advanced melanoma.

## Case report

A 48-year-old man with type 2 diabetes mellitus, nonalcoholic fatty liver disease, and stage IV melanoma (V-Raf murine sarcoma viral oncogene homolog B/neuroblastoma RAS viral oncogene homolog negative) presented to our Dermatology clinic with 2 days of a pruritic, blistering rash.

He was diagnosed with stage IIIC acral lentiginous melanoma in 2018. He underwent a partial amputation of the involved digit and adjuvant nivolumab therapy for 1 year. In 2021, he developed lung metastases. He was treated with nivolumab and ipilimumab, followed by nivolumab monotherapy. Repeat imaging showed worsening lung disease. Given his progression despite anti–programmed cell death protein 1 immunotherapy, he was enrolled in a trial in June 2022 of weekly subcutaneous nemvaleukin alfa monotherapy (NCT04830124).

**Fig 1:**
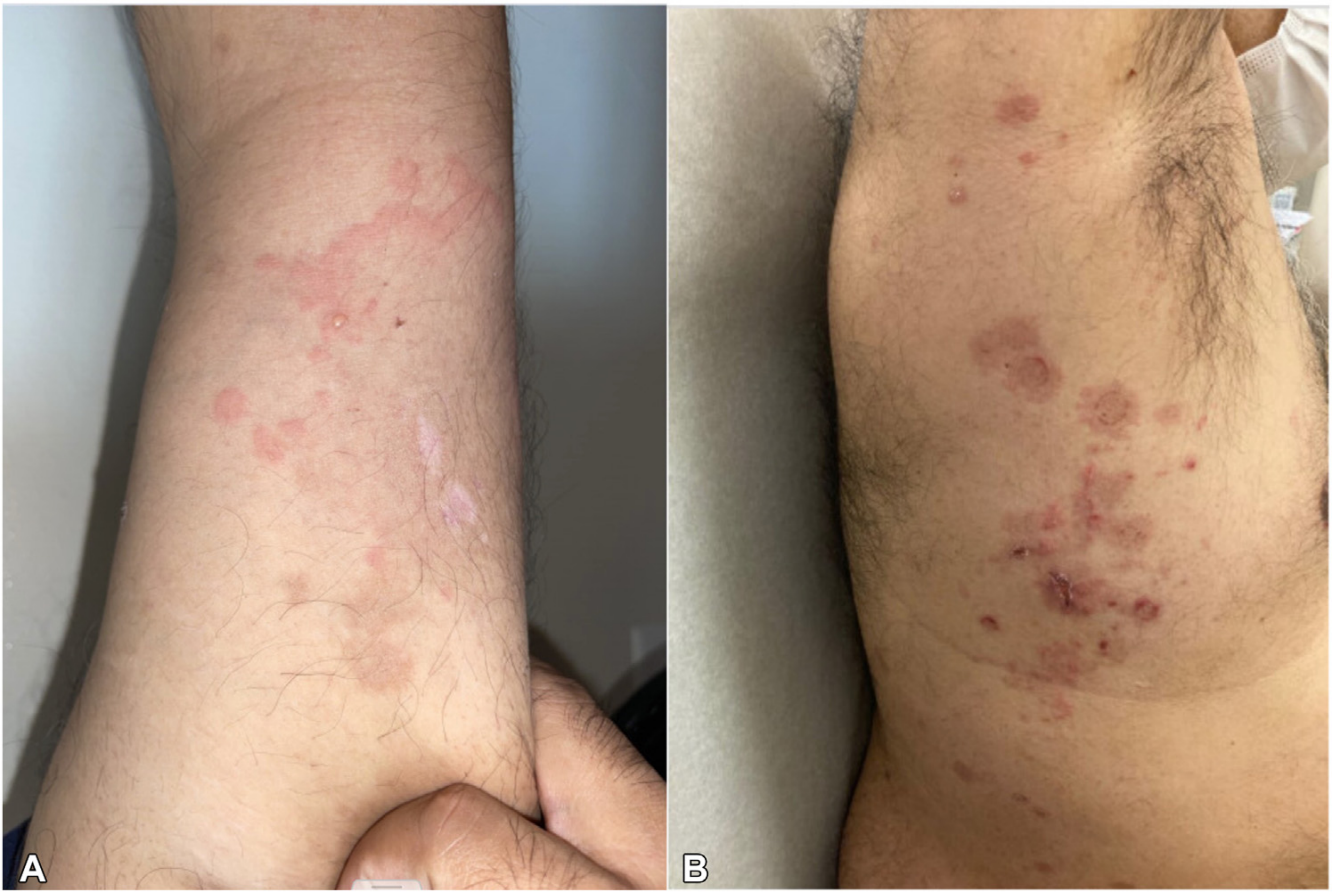
Linear IgA bullous dermatosis. **A,** Ill-defined annular pink plaques on the left upper extremity with scattered vesicles. **B,** Crusted erosions and scattered papules on the right flank.

**Fig 2:**
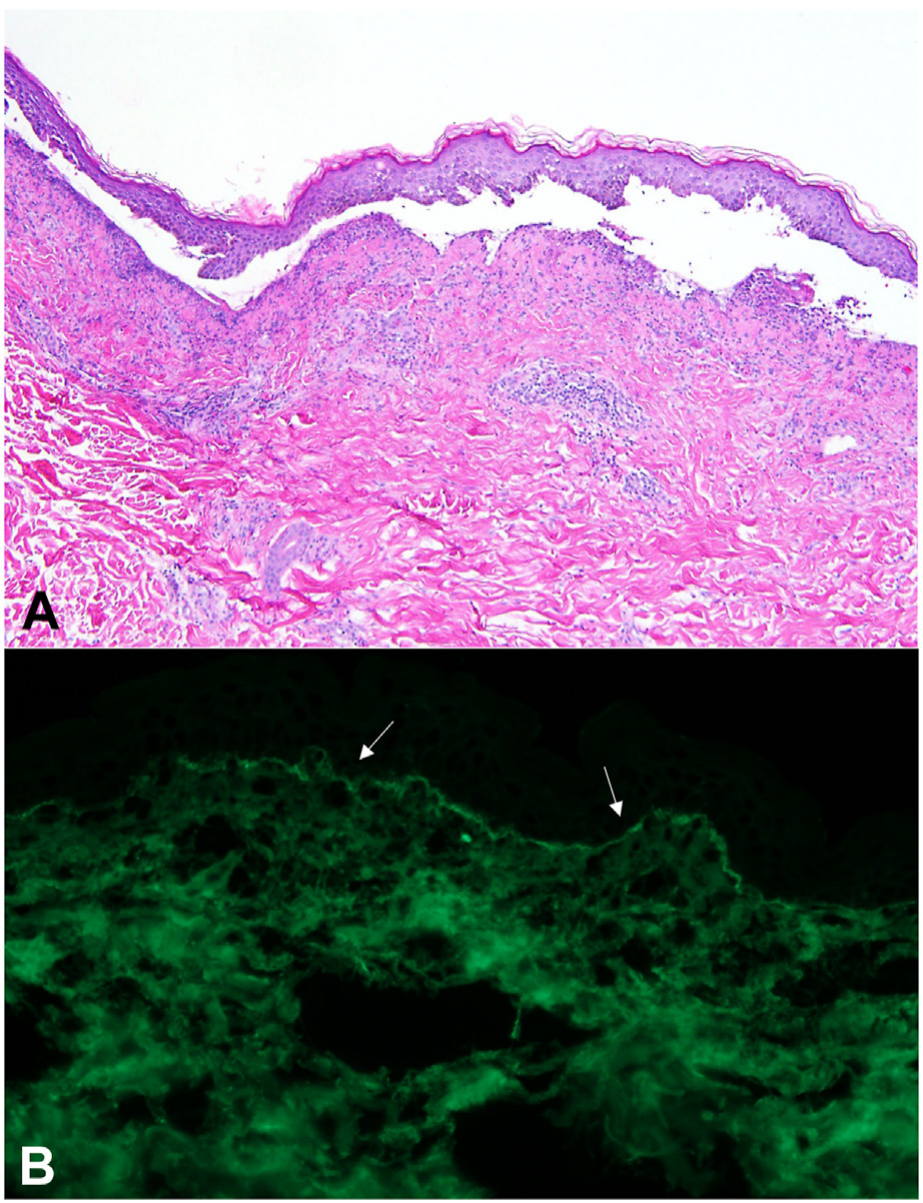
Linear IgA bullous dermatosis. **A,** Hematoxylin and eosin demonstrating subepidermal bullous dermatosis with a superficial perivascular and interstitial neutrophilic infiltrate and collections of neutrophils in the dermal papillae. (Original magnification: 10×.) **B,** Direct immunofluorescence positive for IgA in a linear pattern along the dermoepidermal junction. (Original magnification: ×40.)

He initially tolerated nemvaleukin well, with grade I injection site pain, fatigue, and myalgias resolving within 3 days of each cycle. After the seventh cycle, he developed a rash, keratoconjunctivitis, and dyspnea on exertion. Examination revealed edematous, crusted erosions and scattered papules and pustules on the flanks and buttocks, as well as ill-defined annular plaques with vesicles on the upper extremity (Fig 1). He denied medication changes, sick contacts, or new sexual contacts. Swabs were negative for herpes simplex virus 1/2 and varicella zoster virus. Punch biopsies from the flank revealed subepidermal separation with predominantly neutrophilic infiltrates (Fig 2, *A*). Direct immunofluorescence test revealed linear deposition of IgA along the basement membrane, confirming a diagnosis of LABD (Fig 2, *B*). Nemvaleukin alfa was used.

Over the next 2 weeks, the rash spread to the chest and groin, then improved spontaneously. Nemvaleukin alfa was reinitiated. Two days later, the rash recurred, presenting with erythematous annular plaques on the left upper extremity. He was prescribed clobetasol ointment 0.05% twice daily and dapsone 50 mg/d for 10 days. Nemvaleukin alfa was continued, and the rash resolved over the next 2 weeks. Ultimately, he developed brain metastases, resulting in discontinuation of nemvaleukin.

## Discussion

Drug-induced LABD can have cutaneous presentations ranging from plaques to bullae with positive Nikolsky’s sign, making the diagnosis challenging. IL–2-associated LABD has only been reported once in the English literature in a 1996 case of a patient with metastatic renal cell carcinoma who developed LABD after the second cycle of aldesleukin, a recombinant IL-2 therapy.^2^ The patient was treated successfully with topical and systemic steroids.

Although recombinant IL-2 (aldesleukin) has been US Food and Drug Administration-approved for metastatic melanoma since 1998, its use declined after the introduction of programmed cell death protein 1, V-Raf murine sarcoma viral oncogene homolog B, cytotoxic T lymphocyte associated protein 4, and mitogen-activated protein kinase inhibitors, which revealed superior survival rates and more favorable toxicity profiles.^3^^,^^4^ Nemvaleukin alfa is one of the first engineered (rather than recombinant) IL-2 therapies and has revealed durable antitumor activity in 18.6% of patients with treatment-refractory metastatic melanoma.^5^ It was felt to avoid the severe side effects of high-dose IL-2 therapy by selectively binding to natural killer and CD8^+^ T cells without binding regulatory T cells. To date, the most common side effects of nemvaleukin alfa have been fevers and neutropenia.^5^ There are no previous reports of LABD attributed to nemvaleukin alfa. IL-2 activity in LABD pathogenesis is unclear, with studies suggesting that it may promote B-cell proliferation and antibody production.^2^

Nemvaleukin alfa is being studied in patients with other solid organ tumors (NCT05092360 and NCT02799095). With wider applications of modified IL-2 molecules, it is important for clinicians to be aware of its potential skin toxicities to allow for successful diagnosis and management of these complex and high-need patients.

## Conflicts of interest

None disclosed.
